# Gas Chromatography-Mass Spectrometry (GC-MS) Metabolite Profiling of Citrus limon (L.) Osbeck Juice Extract Evaluated for its Antimicrobial Activity Against Streptococcus mutans

**DOI:** 10.7759/cureus.33585

**Published:** 2023-01-10

**Authors:** Samer S Abed, Kiranmayi P, Khalid Imran, Syed S Lateef

**Affiliations:** 1 Department of Biochemistry, Acharya Nagarjuna University, Guntur, IND; 2 Department of Microbiology, Krupanidhi Degree College, Bengaluru, IND; 3 Wipro Life Sciences Lab, Wipro Limited, Bengaluru, IND

**Keywords:** gc-ms, streptococcus mutans, lemon juice, citrus limon, dental caries

## Abstract

Purpose

The present study aimed to determine the antimicrobial nature of *Citrus limon* juice extract against *Streptococcus mutans* and to identify its metabolic profile by gas chromatography-mass spectrometry (GC-MS) technique.

Materials and methods

The cariogenic bacteria *S. mutans* were procured from Microbial Type Culture Collection and Gene Bank (MTCC), Chandigarh, India, and revived on brain heart infusion (BHI) agar. The *C. limon* (L.) Osbeck fruits were authenticated from the University of Trans-Disciplinary Health Sciences and Technology (TDU), Bengaluru, India. The antibacterial property of lyophilized lemon juice extract (LJE) dissolved in methanol was evaluated against *S. mutans* (MTCC 497) by the agar well diffusion assay. GC-MS technique was employed to identify the volatile metabolite profile of the methanolic LJE sample. The metabolite masses of the respective compounds were identified using the National Institute of Standards and Technology (NIST) library.

Results

The methanolic LJE sample concentration from 5 to 25 mg/ml did not demonstrate antimicrobial activity, while 30 to 100 mg/ml displayed antibacterial activity against *S. mutans*. Chlorohexidine (100 µg/ml) was used as the positive control, while methanol was used as the negative control. Significant antimicrobial metabolites were detected in the methanolic LJE sample by GC-MS analysis. Maleic anhydride, succinic anhydride, 6-Oxa-bicyclo[3.1.0] hexan-3-one, and 3-methyl-2,5-Furandione were the key metabolites identified in the methanolic LJE sample.

Conclusion

The present study reports that *C. limon* is a potential candidate for the pharmaceutical industry as it possesses bioactive compounds demonstrating therapeutic properties. Further investigations are warranted to determine the individual and synergetic effects of identified metabolites in the methanolic LJE sample for its antimicrobial property. Special attention needs to be focussed on translational research for the development of anti-caries products from *C. limon*.

## Introduction

Dental caries is undoubtedly the most common public health concern affecting humans worldwide [1]. Though extensive research has been carried out for decades to combat caries, it still remains unresolved [2]. Hence, the prevention of caries is crucial for managing public health. The principle etiological agent *S. mutans* is most commonly associated with the initiation and development of caries. *S. mutans* plays a vital role in the development of complex, multi-dimensional structures on the tooth enamel and also oral mucosa [3]. It possesses several cariogenic traits that include adhesion to solid surfaces, oral colonization, and enduring an acidic environment [4]. Additionally, *S. mutans* utilize carbohydrates for producing acidic metabolites that result in acidic demineralization of the tooth enamel, and also removal of mineral components, leading to caries [5].

High-risk individuals have received recommendations for a variety of antimicrobial treatments for the prevention of caries, namely biocides like chlorhexidine [6], triclosan [7], and cetylpyridinium chloride [8] as well as antibiotics like vancomycin [9], as these are able to lower mutans streptococci levels. However, the majority of these drugs have broad-spectrum antibacterial effects that suppress even commensal microbiota that is beneficial to health [10]. The emergence of bacterial antibiotic resistance, when exposed to antibiotics, is not only a long-standing phenomenon, but it is also a very challenging issue. Despite the fact that it is a serious concern for human health, adequate attention has not been prioritized towards its prevention. Even though this worrying circumstance is constantly worsening, neither the scientific community nor researchers nor health professionals have taken it into consideration while formulating planning strategies. A global threat is being created by the current crisis of bacterial multidrug resistance, prompting the creation of novel substitutes. As there are not many new antimicrobials on the market, the pharmaceutical industry's "sleeping giants" have a great chance of providing medicines derived from natural sources that can be used to fight the threat posed by antibiotic resistance [11]. As per WHO, nearly 80% of people worldwide rely on herbal remedies to cure some disorders [12], and a minimum of one plant constituent is incorporated in herbal medications [13]. It is crucial to note that extensive research has been done on the bioactivity and bioavailability of phytochemicals [14,15].

*C. limon* is one of the key medicinal plants that belongs to the Rutaceae family [16]. The precise location of *C. limon*'s original natural habitat is not clear [17,18]. *C. limon*'s primary raw material is fruit, specifically juice and essential oil. Despite the fact that fruit of *C. limon* is well-documented for its nutritional benefits, it is crucial to note that contemporary phytotherapy undervalues the fruit's valuable biological activity [17]. Traditional medicine uses for lemon juice include the treatment of common colds, irregular periods, and hypertension [19-21]. Flavonoids are the most significant bioactive compounds in the fruit and juice of *C. limon*, which are responsible for their biological activity. Another significant class of chemicals present in both juice and fruit are phenolic acids [22]. The metabolic profile of the various *C. limon* fruit components, namely the albedo, flavedo, oil glands, pulp, seeds of lemon, and citron, has been previously investigated using high-resolution magic angle spinning nuclear magnetic resonance (HR-MAS NMR) spectroscopy [23]. Most frequently, gas chromatography-mass spectrometry (GC-MS) is employed for the investigation of plant metabolites [24-28]. The gold standard for elucidating phytochemical profiles of plant extracts is GC-MS, due to its great selectivity and sensitivity. The most commonly used bioassay methods for screening the antimicrobial activity of phytochemicals are time-kill assay, agar well diffusion method, and lysis experience [11]. Though *C. limon* juice extract has been earlier reported by various investigators to possess antimicrobial properties against a wide spectrum of microbes, however, there is a lack of studies that are focused to determine *C. limon* Osbeck juice extract’s antimicrobial activity against *S. mutans*, and its metabolic profiling by GC-MS analysis. In this context, the aim of the present investigation was to identify the metabolic profile of *C. limon* juice extract by GC-MS analysis for its antimicrobial activity against *S. mutans*.

## Materials and methods

Collection and identification of *C. limon* fruit

The *C. limon* (L.) Osbeck fruits were collected from the Foundation for Revitalisation of Local Health Traditions (FRLHT), Bengaluru, India. The identification and authentication of the fruit sample were done by Dr. N M Ganesh Babu, Associate Professor, Head, Centre for Herbal Gardens, The University of Trans-Disciplinary Health Sciences and Technology, Bengaluru, India.

Fruit juice preparation of *C. limon*

The collected fruits of *C. limon* were thoroughly washed with sterile distilled water and aseptically cut in two. The fruit juice was aseptically collected by squeezing the fruit. The extracted fruit juice was filtered through a Whatman membrane filter (0.45 µm). The collected lemon juice extract (LJE) was lyophilized. The LJE sample was preserved at 4°C in an air-tight container till further use [29,30].

Revival of *S. mutans* reference strain and antibacterial susceptibility testing

From Microbial Type Culture Collection and Gene Bank (MTCC), Chandigarh, *S. mutans* freeze-dried culture (MTCC 497) was procured. The freeze-dried culture was revived in brain heart infusion (BHI) broth, and later streaked on BHI agar. The streaked plates were anaerobically incubated for 48 hours at 37°C to isolate pure colonies. From the pure culture agar plate, a single isolated colony was aseptically transferred into sterile BHI broth and anaerobically incubated for 24 hours at 37°C.

The agar well diffusion method was used to perform an antibacterial susceptibility test as described by Oikeh et al., with minor modifications [29]. The different concentrations (5, 10, 15, 20, 25, 30, 35, 40, 45, 50, 55, 60, 65, 70, 75, 80, 85, 90, 95, and 100 mg/ml) of LJE samples were prepared in 100% methanol to determine their antibacterial activity. Sterile saline was used to adjust bacterial inoculum, so as to match the 0.5 McFarland turbidity standard. The optimized inoculum was aseptically swabbed on BHI agar (1.7% agar) using a sterile cotton swab. After 15 min, using a sterile cork borer, the BHI agar plates were bored with 7 mm wells in diameter. Thereafter, different concentrations of methanolic LJE samples were aseptically transferred into the agar wells. Methanol and chlorohexidine (100 µg/ml) were the negative and positive control respectively. After the anaerobic incubation for 24 hours at 37°C, the bacterial inhibition zones were measured in millimeters. The reproducibility and reliability of the experiments performed were validated by performing them in triplicates.

GC-MS analysis

The components of the methanolic LJE sample were analyzed by the GCMS-QP2020 NX (Shimadzu Corporation, Kyoto, Japan), which has Rxi-5sil MS gas chromatograph column (30 m x 0.25 mm ID x 0.25 µm thickness) coupled to a single quadrupole mass spectrometer mass detector. The ion source and interface temperature were set at 230°C and 280°C respectively and the scan range is 25-500 m/z. The column temperature was set at 60°C for 5 min and increased to 100°C at the rate of 5°C/min with a 2 min hold time and increased to 150°C at the rate of 15°C/min with a 2 min hold and finally increased to 260°C at the rate of 15°C/min with a 5 min hold time. Helium was used as a carrier gas. The total run time was 30 min. The sample injection volume was 1 µl. The solvent delay time was 4 min and it was injected in a split ratio of 1:10. Masses were identified using the NIST 20 library. Methanol was used as a diluent and profiled for GC-MS analysis. All GC-MS analyses were performed in duplicate to determine their reproducibility and reliability. 

Statistical analysis

The mean and standard deviation of the zone of inhibition at different concentrations of methanolic LJE samples were determined.

## Results

Antibacterial study

**Figure 1:**
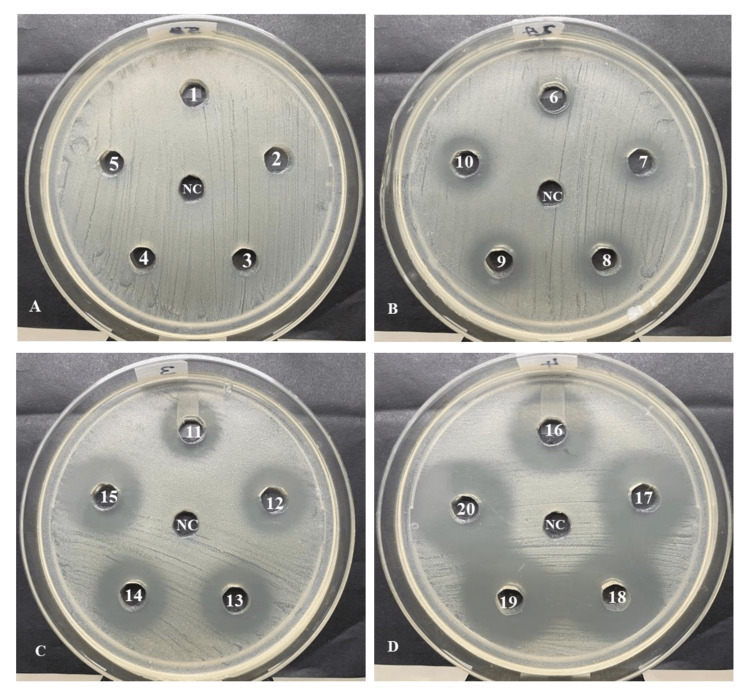
Antimicrobial activity representing the zone of inhibition of lyophilized lemon juice methanolic extract at different concentrations against S. mutans. (A) No. 1 – 5 represents the concentrations from 5 – 25 mg/ml, (B) No. 6 – 10 represents the concentrations from 30 – 50 mg/ml, (C) No. 11 – 15 represents the concentrations from 55 – 75 mg/ml, (D) No. 16 – 20 represents the concentrations from 80 – 100 mg/ml, and (NC) is Negative control.

From the present study results, it is observed that the methanolic LJE sample possesses antimicrobial activity against *S. mutans* strain MTCC 497. The average zone of inhibition representing the antimicrobial activity of the methanolic LJE sample at concentrations ranging from 5 to 100 mg/ml is presented in Table 1. The methanolic LJE sample concentrations from 5 to 25 mg/ml did not demonstrate antimicrobial activity, while 30 to 100 mg/ml displayed antibacterial activity with inhibition zone ranging from 10.67 mm to 24.65 mm, respectively. Among the different methanolic LJE sample concentrations, the highest zone of inhibition (24.65 mm) was observed at 100 mg/ml. The positive control chlorhexidine at a concentration of 100 μg/ml displayed a 32.67 mm zone of inhibition while methanol, the negative control, did not demonstrate antimicrobial activity against *S. mutans*. The representative images of the antibacterial activity of methanolic LJE sample from 5 mg/ml to 100 mg/ml concentrations against *S. mutans* along with negative control are represented in Figure 1A-1D. From the results, it is clearly observed that as the concentration of the methanolic LJE sample increased, the average zone of inhibition also increased.

**Table 1:** Antibacterial activity of lyophilized lemon juice methanolic extract against S. mutans reference strain

Concentration (mg/ml) of lyophilized lemon juice methanolic extract	Average zone of inhibition (mm)	Standard deviation	
			05	0.00	0	
10	0.00	0	
15	0.00	0	
20	0.00	0	
25	0.00	0	
30	10.67	0.58	
35	11.33	0.58	
40	12.67	0.58	
45	13.67	0.58	
50	15.00	1.00	
55	15.33	0.58	
60	17.33	1.15	
65	17.00	1.00	
70	17.67	0.58	
75	19.33	0.58	
80	21.33	0.58	
85	22.33	0.58	
90	22.00	1.00	
95	22.33	0.58	
100	24.65	0.58	
Positive control	32.67	0.58	
Negative control	0.00	0.00	

GC-MS analysis

**Figure 2:**
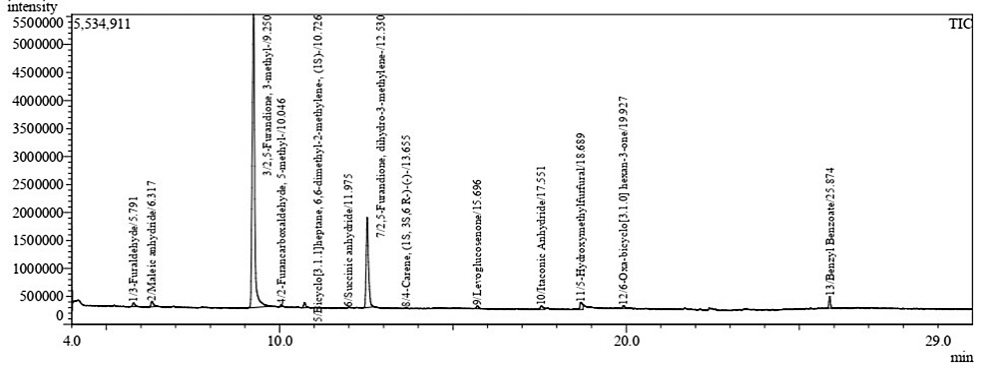
Gas Chromatography-Mass Spectrometry chromatogram of lyophilized lemon juice methanolic extract

The GC-MS metabolite profile of the methanolic LJE sample is presented in Figure 2. As benzyl benzoate was detected in both the blank as well as methanolic LJE sample profiles, it was excluded from the analysis. Significant metabolites were identified in the GC-MS profile of the methanolic LJE sample as listed in Table 2. The different metabolites identified by GC-MS analysis belong to the class/origin furans, aryl aldehydes, terpene, bicyclic mono-terpenoids, citric acid derivative, cellulose and sugar derivatives, and a fairly new compound 6-Oxa-bicyclo[3.1.0]hexan-3-one [31] was also detected (Table 2). Among the metabolites identified in the methanolic LJE sample, two metabolite peaks, namely 2,5-Furandione, 3-methyl- (origin- derived from citric acid) and 2,5-Furandione, dihydro-3-methylene (class- Furan) at retention time 9.250 and 12.530, respectively were found to be predominant in the GC-MS profile. 

**Table 2:** Metabolite profile of lyophilized lemon juice methanolic extract identified by Gas Chromatography-Mass Spectrometry analysis

Sl. No.	Metabolites Identified	Retention Time (min)	CAS No.	Metabolite Class/ Origin
1	3-Furaldehyde	5.791	498-60-2	Furan
2	Maleic anhydride	6.317	108-31-6	Furan
3	2,5-Furandione, 3-methyl-	9.250	616-02-4	Derived from Citric acid
4	2-Furancarboxaldehyde, 5-methyl-	10.046	620-02-0	Aryl Aldehydes
5	Bicyclo [3.1.1] heptane, 6,6-dimethyl-2-methylene-, (1S)	10.726	18172-67-3	Terpene
6	Succinic anhydride	11.975	108-30-5	Furan
7	2,5-Furandione, dihydro-3-methylene-	12.530	2170-03-8	Furan
8	4-Carene, (1S,3S,6R-)-(-)-	13.655	5208-49-1	Bicyclic monoterpenoids
9	Levoglucosenone	15.696	37112-31-5	Derived from cellulose
10	Itaconic anhydride	17.551	2170-03-8	Pyrolysis of citric acid
11	5-Hydroxymethylfurfural	18.689	67-47-0	Derived from sugars
12	6-Oxa-bicyclo[3.1.0] hexan-3-one	19.927	74017-10-0	New compound [31]

## Discussion

The current study was primarily focused on *S. mutans*, as it is the principle etiological agent involved in caries. In the present study, a significant antimicrobial activity of the methanolic LJE sample against *S. mutans *was observed at concentrations ranging from 30 - 100 mg/ml (Table 1). The results clearly demonstrate that the methanolic LJE sample is a potential anti-*S. mutans* agent. Chlorhexidine has been widely used in dentistry as a gold-standard antiseptic for more than four decades and is used in various oral healthcare products [32]. The risk of chlorhexidine resistance is a global concern, and there is a lack of awareness among the public.

Previously investigators have reported that the juice of *C. limon* demonstrated intermediate (11 to 15 mm) and sensitive (16 to 19 mm) zone of inhibition against enterotoxin *Escherichia coli* for concentrations ranging from 500 to 800 mg/ml and 900 to 1000 mg/ml respectively [33]. Singh et al. reported that *Shigella flexneri* were highly sensitive to *C. limon* juice followed by *Citrobacter* species, *Staphylococcus epidermidis,* and *Salmonella typhi* [16]. The zone of inhibition of bacterial species varied from a minimum of 6.0 mm to a maximum of 15.2 mm for concentrations ranging from 100 to 1000 µg/disc. In another study, the *Citrus limonum* extract displayed antibacterial activity against *Bacillus subtilis*, *Pseudomonas aeruginosa*, *Proteus* spp., *E. coli*, *Staphylococcus aureus*, *Salmonela typhi* and *Salmonella kintambo* [30]. The Gram-positive bacteria, namely *Enterococcus faecalis* and *S. aureus,* were comparatively more sensitive than the Gram-negative bacteria that include *E. coli*, *Salmonella* spp., and *P. aeruginosa*. A lower antibacterial susceptibility pattern was demonstrated in Gram-negative bacteria when compared with Gram-positive bacteria [29]. 

Earlier, investigators have reported that the antimicrobial property of lemon juice may be attributed to the prevalence of organic acids, secondary metabolites, vitamins, and their respective interactions with each other [30]. The naturally occurring organic acids in vegetables and fruits inhibit microbial growth. Citric, acetic, maleic, tartaric, sorbic, and succinic acids are the primary organic acids that occur naturally in vegetables and fruits. These acids are responsible for a reduction in pH, depression of the inner pH of the organisms by un-dissociated acid molecule ionization, or substrate transport system breakdown by altering the permeability of the cell membrane [34].

From the GC-MS metabolite profile of the methanolic LJE sample, 12 significant metabolites were identified (Table 2) that might be involved in antimicrobial activity against *S. mutans*. Among the metabolites identified, maleic anhydride, succinic anhydride, 6-Oxa-bicyclo[3.1.0]hexan-3-one, and 3-methyl-2,5-Furandione may be involved in antimicrobial activity against *S. mutans*. Additional techniques such as liquid chromatography coupled with a high-resolution mass spectrometer (LC-HRMS) can be used to further increase the number of metabolite identification.

Sugars are commonly reported to be part of flavanone glycoside commonly found in citrus fruits. In GC-MS, dehydrated sugar metabolites are most likely formed due to the heated source of the GC-MS inlet. The levoglucosenone and 5-Hydroxymethylfurfural metabolites detected in the methanolic LJE sample are formed from the dehydration of sugars. Due to thermal dehydration in GC-MS, maleic and succinic acid are detected in the form of anhydrides [35,36], and in our study, maleic and succinic anhydrides are detected in the GC-MS profile of the methanolic LJE sample attributing to the presence of the respective acids. Maleic and succinic acid have been earlier reported to possess antimicrobial properties, while 6-Oxa-bicyclo[3.1.0]hexan-3-one and 3-methyl-2,5-Furandione metabolites were detected in plant materials that demonstrated strong antimicrobial activities in previously reported studies [31,37-42]. Maleic acid and lactic acid displayed higher antimicrobial activity than citric acid against *E. coli*, *Listeria monocytogenes*, and *Salmonella gaminara* in one of the previous studies [37]. Investigators have also earlier reported that 7% of maleic acid possess antifungal and antibacterial activities against *Candida albicans*, *E. faecalis*, and *S. aureus*, at different time intervals [38]. Additionally, maleic acid has been reported to disrupt *E. faecalis* biofilms at low concentrations, namely 0.88% and 0.11%, at exposure to 30 seconds and two minutes, respectively [39]. Investigators have reported that succinic acid (1 mg/disc) displayed a strong antimicrobial activity against New Delhi metallo-β-lactamase 1 (NDM-1) *E. coli*, greater than colistin 10 (µg/disc) and have proposed that organic acids can be employed to inhibit bacterial growth and is an alternative source of antibiotic usage. It is recommended that the combination therapy of organic acids and antibiotics could be a safe method for clinical applications in combating NDM-1 *E. coli* [40]. In one of the earlier studies, the ethyl acetate fraction of pickled and dried *Brassica juncea* Coss. var. *foliosa* Bailey demonstrated strong antibacterial activity against *S. aureus* and *Pseudomonas fluorescens*. Succinic acid was the prime component in pickled and dried *Brassica juncea* Coss. var. *foliosa* Bailey, responsible for the antimicrobial activity, identified by UV-visible spectrophotometry (UV-Vis), high-performance liquid chromatography (HPLC), GC-MS, Fourier-transform infrared spectroscopy (FT-IR), and nuclear magnetic resonance (NMR) techniques. It is also reported that succinic acid, might be involved by disrupting the cell membrane along with intracellular structure by leaking the cell components [41]. Previously investigators have reported that *Cinnamomum zeylanicum* methanolic extract possesses remarkable antibacterial and antifungal activity; 6-Oxa-bicyclo[3.1.0]hexan-3-one was detected as one of the phytochemical constituents of the extract and its pharmacological action is not reported as it is a new chemical compound [31]. The GC-MS profile of *Pelargonium peltatum*, which displays antimicrobial and antioxidant activities, possesses 3-methyl-2,5-Furandione in the freeze-dried extract of leaf and flower [42]. Based on the earlier findings and the results of the present study, further investigations need to be carried out on maleic acid, succinic acid, 6-Oxa-bicyclo[3.1.0]hexan-3-one and 3-methyl-2,5-Furandione for its individual and synergetic antimicrobial activity against *S. mutans*.

In oral healthcare products, many synthetic compounds have been used for better oral health management, but unfortunately, these compounds have serious adverse effects and also develop antibiotic resistance in bacteria. The incorporation of naturally occurring compounds that possess antimicrobial activity, especially natural organic acids is an important factor in the development of anti-caries products. To summarize the results, the present study demonstrates that *C. limon* is a promising candidate for further studies of caries prevention and management. 

The limitation of the present study is that GC-MS can be employed only for broad-spectrum metabolite screening, and metabolite confirmation studies, and also the technique cannot analyze substances that are non-volatile in nature, thermally sensitive, or polar [43]. Another limitation may be that the non-volatile metabolites of the LJE sample might also be involved in the antimicrobial activity. 

## Conclusions

The biological activities of *C. limon* have been studied extensively and reported to possess a wide range of applications in phytopharmacology. In the present study, key metabolites, maleic anhydride, succinic anhydride, 6-Oxa-bicyclo[3.1.0]hexan-3-one and 3-methyl-2,5-Furandione were detected in GC-MS chromatogram, which has been earlier demonstrated to possess antimicrobial and anti-biofilm activities. Further investigation needs to be carried out to identify the individual or combinatorial effects of the other metabolites identified in this study that are involved in antimicrobial activity. Additionally, further formulation studies are recommended to be carried out using these naturally available metabolites from *C. limon* for the development of anti-caries products. The usage of natural anti-caries products in oral health may drastically reduce the spread of antibiotic resistance among bacteria and also aid in better caries management.
